# Disrupted Neutrophil and Myeloid Cell Homeostasis and Effector Dysfunction Drive Vasculopathy in Idiopathic Inflammatory Myopathies

**DOI:** 10.3390/cells15121062

**Published:** 2026-06-10

**Authors:** Daniel Alberto Carrillo-Vázquez, Beatriz Alcalá-Carmona, Jennifer Tiaré Balderas Miranda, Yatzil Reyna-Juárez, María José Ostos-Prado, Fabiola Cassiano-Quezada, Samuel Govea-Peláez, Nancy R. Mejía-Domínguez, Guillermo Juárez-Vega, Karina Santana-De Anda, Jiram Torres-Ruiz, Diana Gómez-Martín

**Affiliations:** 1 Department of Immunology and Rheumatology, Instituto Nacional de Ciencias Médicas y Nutrición Salvador Zubirán, Vasco de Quiroga 15, Tlalpan, Mexico City 14080, Mexico; daniel.carrillov@incmnsz.mx (D.A.C.-V.); beatriz.alcalac@gmail.com (B.A.-C.); jennifertbalderasm@gmail.com (J.T.B.M.); yatzilreyna@gmail.com (Y.R.-J.); fabiolacassianoq@gmail.com (F.C.-Q.); samuel.govea@gmail.com (S.G.-P.); karina.santana@incmnsz.mx (K.S.-D.A.); jiram.torresr@incmnsz.mx (J.T.-R.); 2 Department of Immunology, Escuela Nacional de Ciencias Biológicas, Instituto Politécnico Nacional, Mexico City 07738, Mexico; 3 Department of Internal Medicine, Instituto Nacional de Ciencias Médicas y Nutrición Salvador Zubirán, Vasco de Quiroga 15, Tlalpan, Mexico City 14080, Mexico; marix.ostos@gmail.com; 4 Red de Apoyo a La Investigación, Coordinación de Investigación Científca, Universidad Nacional Autónoma de México, Mexico City 14080, Mexico; nmejia@cic.unam.mx (N.R.M.-D.); guillermovega@cic.unam.mx (G.J.-V.)

**Keywords:** vasculopathy, neutrophils, LDG, MDSC, Arginase-1, PDL, rTEM, CD177, phagocytosis

## Abstract

**Highlights:**

**What are the main findings?**

- The expansion of low-density granulocytes and Arginase-1^+^/PDL1^+^ MDSCs quantitatively characterizes vasculopathy associated with IIM.
- There is a reduction in phagocytic capacity and respiratory burst formation in neutrophils from patients with vasculopathy.

**What are the implications of the main findings?**

- Decreased naïve neutrophils and increased activated CD177^+^ neutrophils are independent risk factors for developing the vasculopathic phenotype in IIM.
- Patients with IIM with vasculopathic features had an imbalance between proinflammatory and regulatory neutrophil subsets, alongside persistent functional impairment, regardless of disease activity status.

**Abstract:**

Background: Neutrophils play a role in idiopathic inflammatory myopathies (IIMs), especially in vasculopathic manifestations. While neutrophil extracellular traps (NETs) and low-density granulocytes (LDGs) have been described, the functional relevance of other subsets—such as naïve neutrophils, reverse transendothelial migration neutrophils (rTEM), myeloid-derived suppressor cells (MDSCs), and regulatory neutrophils—and their association with vasculopathy remains unknown. Objective: We aimed to characterize the phenotypic and functional profile of neutrophil subsets and their association with vasculopathic manifestations in IIM, adjusting for disease activity. Methods: We conducted a cross-sectional, single-center study including 59 IIM patients diagnosed by muscle biopsy and fulfilling 2017 ACR/EULAR criteria. Flow cytometry was used to immunophenotype myeloid subsets, and functional assays assessed phagocytosis and respiratory burst. Patients were stratified by clinical activity and presence of vasculopathy. Results: Vasculopathic patients showed expansion of LDGs (*p* = 0.0092), granulocytic and monocytic MDSCs expressing Arginase-1 (*p* = 0.0078, *p* = 0.0003) and PD-L1 (*p* = 0.0258, *p* = 0.0087), and rTEM neutrophils (*p* = 0.0775). In contrast, they exhibited a reduction in naïve neutrophils (*p* = 0.0004), phagocytosis (*p* < 0.0001) and respiratory burst (*p* = 0.0006). Multivariate analysis identified naïve neutrophils and activated CD177^+^ neutrophils as independent predictors of vasculopathy. A positive correlation between activated and naïve neutrophils were observed in patients with vasculopathic features (r = 0.43; *p* < 0.05). Conclusions: IIM patients with vasculopathic features display a distinct immune profile characterized by an imbalance between proinflammatory and regulatory neutrophil subsets, alongside persistent functional impairment.

## 1. Introduction

Idiopathic inflammatory myopathies (IIMs) are multisystemic autoimmune diseases characterized by myositis with proximal muscle weakness, accompanied by hallmark cutaneous manifestations [1]. These disorders are marked by immune-mediated vascular injury, and overexpression of type I interferon (IFN-I)-related genes, particularly in the skin, lung, and muscle [2,3,4,5], driving microvascular dysfunction with disruption of endothelial permeability.

Vasculopathy in IIM represents a continuum ranging from overt inflammatory vasculitis to non-inflammatory occlusive microvasculopathy [6]. Regardless of the spectrum, a shared pathogenic outcome emerges—an imbalance between angiogenic and profibrotic factors—resulting in chronic hypoxia and myofibroblast differentiation. This is accompanied by endothelial activation, reflected by upregulation of intercellular adhesion molecule-1 (ICAM-1) and vascular cell adhesion protein 1 (VCAM-1), features frequently observed in dermatomyositis and antisynthetase syndrome, where the IFN-I signature, particularly IFN-κ, is markedly upregulated [7,8].

Distinct serological subtypes show specific clinical associations with vasculopathic features: anti-transcription intermediary factor 1-gamma (TIF-1γ) with erythroderma and extensive psoriasiform plaques; anti-melanoma differentiation-associated protein 5 (MDA5) with necrotic ulcers and interstitial lung disease (ILD); and anti-nuclear matrix protein 2 (NXP2), anti-Ku, anti-polymyositis/scleroderma (PMScl), or anti-Ro52 (TRIM21) with calcinosis (up to 10%), Raynaud’s phenomenon (25%), livedo reticularis, and leukocytoclastic vasculitis [1,9]. Despite this, current activity assessment tools do not include vasculopathy as a domain for therapeutic decision-making [10].

Among immune cell players, neutrophils have emerged as key effectors in vasculopathy, mediating phagocytosis, reactive oxygen species (ROS) generation, and neutrophil extracellular trap (NET) formation [11]. NETs induce vasculopathy, while amplifying IFN-I production [12,13]. Anti-MDA5 antibodies can induce NETosis in vitro [14]. Our group has reported an expansion of low-density granulocytes (LDGs) in anti-MDA5-positive patients [15]. Contrary to the classical view that neutrophils are short-lived terminal cells, studies have demonstrated a lifespan of up to 5.4 days [16]. The migration from sites of inflammation back into the vascular system was described as neutrophil reverse transendothelial migration (rTEM) [17]. This process occurs due to an active desensitization to the chemotactic gradient by TNF-alpha, and the expression of JAM-C [18], as well as a unique phenotype characterized by CD54high, CXCR1low expression [17]. Two main features define this granulocyte subtype: the ability to be rescued from apoptosis due to lower caspase-3 expression; and being a tissue-experienced subpopulation with increased superoxide production [17]. The functional role of this neutrophil subset remains uncertain; however, reverse migration can facilitate the dissemination of previously primed neutrophils to other organs, perpetuating systemic inflammation in contexts of sustained chronic inflammation [19,20,21].

Our research group was the first to report the association between myeloid-derived suppressor cells (MDSCs), disease activity and cumulative damage in IIMs. MDSCs may act as promoters rather than protectors of tissue damage, as their immature phenotype impairs their ability to counteract the inflammatory response [22].

In summary, the differential role of neutrophil subpopulations—proinflammatory (LDG, rTEM) and immunomodulatory (naïve, regulatory), as well as MDSCs—in the pathophysiology of vasculopathic manifestations in IIMs remains unknown. Moreover, there is no clear evidence regarding their specific functions (phagocytosis and respiratory burst) or their correlation with the vasculopathic clinical phenotype, which prompted the present study.

## 2. Materials and Methods

A cross-sectional, single-center study was conducted including 59 patients from the National Institute of Medical Sciences and Nutrition Salvador Zubirán (INCMNSZ) in Mexico City between April and December 2024, diagnosed with idiopathic inflammatory myopathy (IIM), confirmed by muscle biopsy and classified according to the 2017 ACR/EULAR criteria for dermatomyositis [23], clinically amyopathic dermatomyositis based on Sontheimer criteria [24] and antisynthetase syndrome according to Connors criteria [25]. All patients belonged to the institutional cohort MYOsitis Translational Research Cohort Salvador Zubirán (MYOTReCSZ).

Patients were recruited from the rheumatology outpatient clinic and emergency or inpatient hospitalization wards. A medical evaluation was performed, including the two disease activity scales from the IMACS group: manual muscle testing (MMT-8) and the Cutaneous Dermatomyositis Disease Area and Severity Index (CDASI). Muscle enzyme levels were assessed (CPK, aldolase, alanine aminotransferase, aspartate aminotransferase, LDH) [26]. The protocol was approved by the Ethics Committee of the INCMNSZ (reference number 2152). All participants provided written informed consent voluntarily.

Adults (>18 years) with confirmed IIM were enrolled and classified into four groups according to disease activity—defined as MMT-8 < 136 and/or CDASI > 6—and presence of vasculopathy, operationally defined as interstitial lung disease (ILD) on HRCT or PFTs, calcinosis (≥2 lesions >2 mm) [27], active digital ulcers (≥1 ischemic ulcer with recent worsening, >1 within 6 months, or >2 within 12 months) [28] or periungual lesions. Inactive patients have shown no clinical activity ≥6 months (MMT-8 > 136 and/or CDASI < 6) without vasculopathy. Final groups were: active + vasculopathy (*n* = 7), active – vasculopathy (*n* = 7), inactive + vasculopathy (*n* = 23), and inactive – vasculopathy (*n* = 22). Exclusion criteria included cancer, chronic renal/hepatic disease, acute/chronic infections, pregnancy, postpartum, or overlap syndromes.

**Study variables:** Demographic variables included age and sex assigned at birth. Clinical variables encompassed myopathy type, prior immunosuppressive therapy, MMT-8 score, extramuscular activity, CDASI activity and damage indices, myositis damage index (MDI), and vasculopathy features (ILD, digital ulcers, calcinosis, periungual lesions). Immunological variables comprised quantification of: regulatory neutrophils (CD62Llow, CD16hi, CD184−, CD11bhi, CD11chi, CD54hi), mature granulocytic MDSCs (HLA-DR−, CD11b+, CD33dim, CD14−, CD66b+, Arginase-1 ±, PD-L1 ± [Arg1 and PD-L1 MFI]), immature granulocytic MDSCs (HLA-DR−, CD11b+, CD33dim, CD14−, CD16−, Arginase-1 ±, PD-L1 ± [Arg1 and PD-L1 MFI]), monocytic MDSCs (HLA-DR−, CD11b+, CD33hi, CD14+, CD16−, Arginase-1 ±, PD-L1 ± [Arg1 and PD-L1 MFI]), low-density granulocytes (LDGs) (CD66b+, CD14−), activated neutrophils (CD15+, CD16+, CD177+ [CD177 MFI]), rTEM neutrophils (CD15+, CD16hi, CD54hi, CXCR1low), and naïve neutrophils (CD15+, CD16hi, CD54low, CXCR1hi).

Peripheral venous blood samples (20 mL) were collected from all included patients, and clinical data were extracted from electronic medical records. The evaluation of peripheral blood cell subpopulations was carried out as follows:

### 2.1. Determination of Granulocytic and Monocytic Subpopulations in Peripheral Blood

Peripheral blood was diluted 1:1 with phosphate-buffered saline (PBS) and layered over Ficoll-Paque PLUS (GE Healthcare™, Chicago, IL, USA) for density gradient separation. After centrifugation (25 min, 2500 rpm, RT), the mononuclear cell (MNC) layer was collected, washed twice (PBS, 1600 rpm, 10 min), and resuspended in RPMI 1640 (Gibco™, Carlsbad, CA, USA) with 10% fetal bovine serum (FBS) and 1% antimycotic antibiotic. Cell counts and viability were determined by trypan blue exclusion using a Neubauer chamber.

Neutrophils were obtained by dextran sedimentation (20% dextran, Alfa Aesar™, Ward Hill, MA, USA), followed by erythrocyte lysis (0.2% NaCl, 5 min) and osmotic restoration (1.8% NaCl). After centrifugation (10 min, 1600 rpm), cells were resuspended in phenol red-free RPMI for counting and viability assessment.

**Figure 1:**
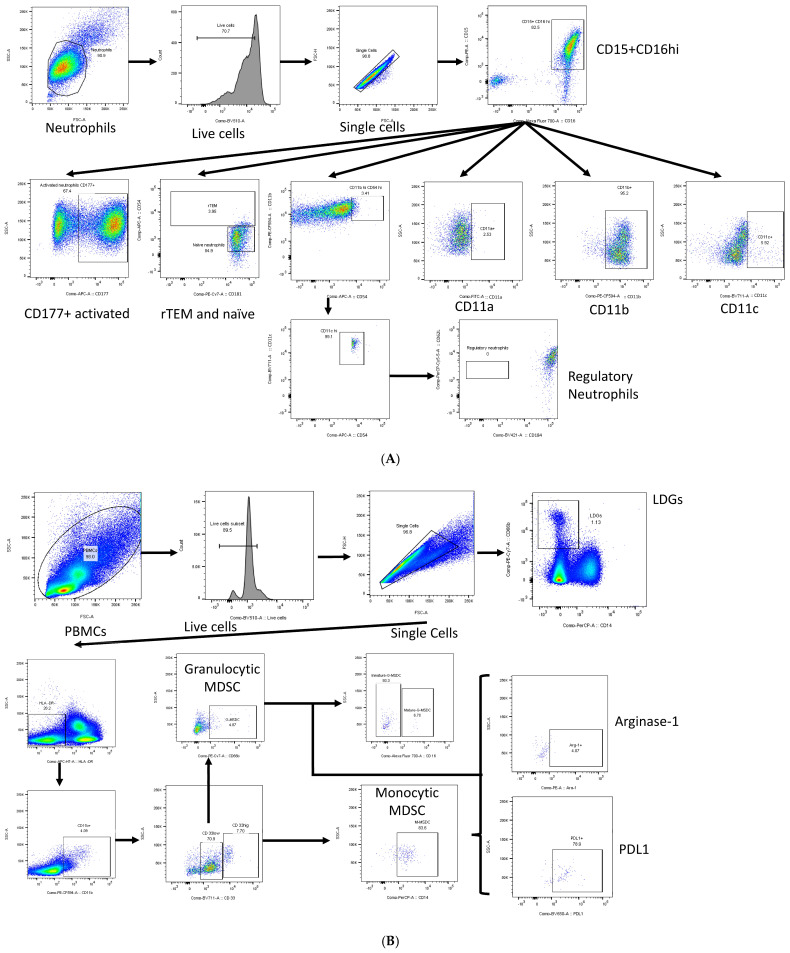
Gating strategy employed for the immunophenotypic characterization of peripheral blood neutrophil subpopulations and myeloid-derived suppressor cells (MDSCs). (**A**) Identification of activated neutrophils, reverse transendothelial migrating neutrophils (rTEM), naïve neutrophils, regulatory neutrophils, and assessment of integrin expression markers CD11a, CD11b, and CD11c. (**B**) Characterization of low-density granulocytes (LDGs), mature and immature granulocytic MDSCs, and monocytic MDSCs, including their intracellular expression of Arginase-1 and programmed death-ligand 1 (PDL1).

For flow cytometry, 3 × 10^6^ MNCs or neutrophils were washed with PBS, stained with Zombie Aqua™ (BioLegend, San Diego, CA, USA, 1.5 μL, 15 min), washed twice in PBS + 5% FBS, and blocked with Fc Block (3 μL, 10 min). Cells were incubated (30 min, RT, dark) with 2.5 μL each of CD15-PE, CD62L-PerCP/Cy5.5, CD16-AF700, CD11a-FITC, CD11b-PE/Dazzle, CD11c-BV711, CXCR1-PE/Cy7, ICAM-1-APC, CXCR4-BV421, CD177-APC, HLA-DR-APC/H7, CD33-BV711, CD14-PerCP, PDL1-BV650, and CD66b-PE/Cy7 (BioLegend™, San Diego, CA, USA). Cells were washed twice and fixed in 1% paraformaldehyde (300 μL). For MDSC and low-density granulocyte panels, cells were fixed/permeabilized (BD Cytofix/Cytoperm, 250 μL, 30 min, 4 °C), washed, and stained intracellularly with anti-arginase-1-PE (5 μL, 30 min, 4 °C). Data were acquired on a BD LSRFortessa (100,000 events/panel) and analyzed in FlowJo v10.6. Gating strategy is shown in Figure 1.

### 2.2. Functional Assessment of Phagocytosis and Respiratory Burst

Neutrophils were incubated with pHrodo Red E. coli BioParticles (Thermo Fisher, Waltham, MA, USA) for 30 min at 37 °C. Phagocytosis was expressed as the percentage of pHrodo-positive dextran-isolated neutrophils, with mean fluorescence intensity (MFI) indicating phagocytic capacity. Absolute counts were derived from same-day complete blood counts (CBCs). Respiratory burst was measured using the Neutrophil Respiratory Burst Assay Kit (Cayman Chemical, Ann Arbor, MI, USA). Neutrophils were loaded with dihydrorhodamine 123, stimulated with phorbol 12-myristate 13-acetate for 45 min at 37 °C, and analyzed for DHR-positive proportions and MFI. Absolute counts were normalized to neutrophil counts from same-day CBCs.

### 2.3. Statistical Analysis

Categorical variables were compared by Fisher’s exact test, and quantitative data are expressed as medians (IQR). Variance homogeneity was assessed by Brown–Forsythe and Bartlett’s tests. Non-parametric data were analyzed with Kruskal–Wallis and Dunn’s post hoc tests, or with Mann–Whitney U and Wilcoxon signed-rank tests for paired samples. Logistic regression identified myeloid subpopulations associated with vasculopathy, reporting ORs (95% CI). Correlation matrices explored relationships among subpopulations. Statistical significance was set at *p* < 0.05. Statistical analyses were performed with the support of GraphPad Prism™ (Boston, MA, USA) and R software (R statistical software V4 1.2 R Core Team 2021).

## 3. Results

A total of 59 IIM patients were included, predominantly female (81.3%) with a median age of 52.5 years. As expected, patients with active disease but without vasculopathy had significantly lower MMT-8 scores (84 [42–148] vs. 139 [133–149], 150 [150–150], 150 [150–150]; *p* = 0.0011) and elevated muscle enzymes including CPK, aldolase, and LDH compared to the other groups. Ferritin levels were also higher in this group (501 ng/mL [120–1311] vs. 173.8 [33.7–253.6], 49 [18–95.7], 54.2 [23.65–95.75]; *p* = 0.004). Inactive patients with vasculopathy showed a lower absolute neutrophil count than inactive patients without vasculopathy (2910/μL [2555–4365] vs. 4290/μL [3303–5283]; *p* = 0.0129). No differences were noted in CDASI scores or other laboratory features (Table 1). Among vasculopathic patients, anti-MDA5 (23.7%) and anti-Ro52 (11.9%) antibodies were most frequent. Immunosuppressive regimens did not differ among groups (Table 1).

**Figure 2:**
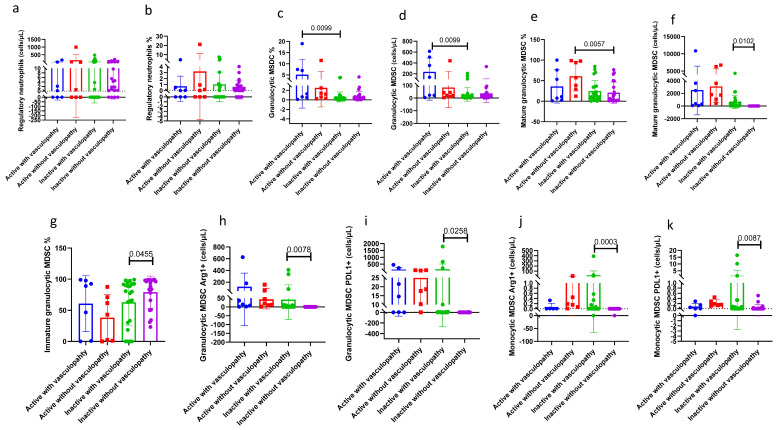
Distinct MDSC phenotypic shifts and checkpoint marker upregulation in vasculopathic IIM. No significant differences were found in regulatory neutrophils between patients with or without vasculopathy (28.8 cells/µL [0–471] vs. 36.38 cells/µL [0.26–192.8]; *p* = 0.476). (**a**,**b**). Patients with vasculopathy showed increased mature granulocytic myeloid-derived suppressor cells (MDSCs) (285.8 cells/μL [0.53–831.4] vs. 1.196 cells/μL [0.223–7.49]; *p* = 0.0102), but decreased immature granulocytic MDSCs (80% [30–92%] vs. 93.7% [58.45–98.3%]; *p* = 0.045). (**c**–**g**). Additionally, higher absolute counts of both granulocytic (2.52 cells/μL [0.037–9.35] vs. 0.024 cells/μL [0.0042–0.114]; *p* = 0.078) and monocytic (0.0072 cells/μL [0.0006–0.245] vs. 0.0000013 cells/μL [0.00000004–0.0000953]; *p* = 0.0003) MDSCs expressing Arginase-1^+^ and PDL1^+^ granulocytic (1.939 cells/μL [0.026–8.432] vs. 0.055 cells/μL [0.009–0.200]; *p* = 0.0258), and monocytic (0.017 cells/μL [0.00007–0.149] vs. 0.000025 cells/μL [0.0000003–0.014]; *p* = 0.0087) were observed. (**h**–**k**). Statistical analyses used included Kruskal–Wallis with Dunn’s multiple comparisons and Mann–Whitney U tests.

Regarding neutrophil subpopulations, regulatory neutrophil counts did not differ between vasculopathy and non-vasculopathy groups (28.8 cells/μL [0–471] vs. 36.38 cells/μL [0.26–192.8]; *p* = 0.476) (Figure 2a,b). Patients with vasculopathy displayed a significant expansion of mature granulocytic myeloid-derived suppressor cells (MDSCs) (285.8 cells/μL [0.53–831.4] vs. 1.196 cells/μL [0.223–7.49]; *p* = 0.0102), with no differences in total granulocytic MDSCs (Figure 2c,d). Conversely, inactive patients with vasculopathy had fewer immature granulocytic MDSCs by percentage (80% [30–92%] vs. 93.7% [58.45–98.3%]; *p* = 0.045), but absolute counts were similar (Figure 2e–g). Functionally, vasculopathic patients showed increased Arginase-1^+^ (2.52 cells/μL [0.037–9.35] vs. 0.024 cells/μL [0.0042–0.114]; *p* = 0.078) and PDL1^+^ granulocytic MDSCs (1.939 cells/μL [0.026–8.432] vs. 0.055 cells/μL [0.009–0.200]; *p* = 0.0258) (Figure 2h,i). Similarly, monocytic MDSCs had elevated Arginase-1^+^ (0.0072 cells/μL [0.0006–0.245] vs. 0.0000013 cells/μL [0.00000004–0.0000953]; *p* = 0.0003) and PDL1^+^ counts compared to activity-matched controls without vasculopathy (0.017 cells/μL [0.00007–0.149] vs. 0.000025 cells/μL [0.0000003–0.014]; *p* = 0.0087) (Figure 2j,k).

**Figure 3:**
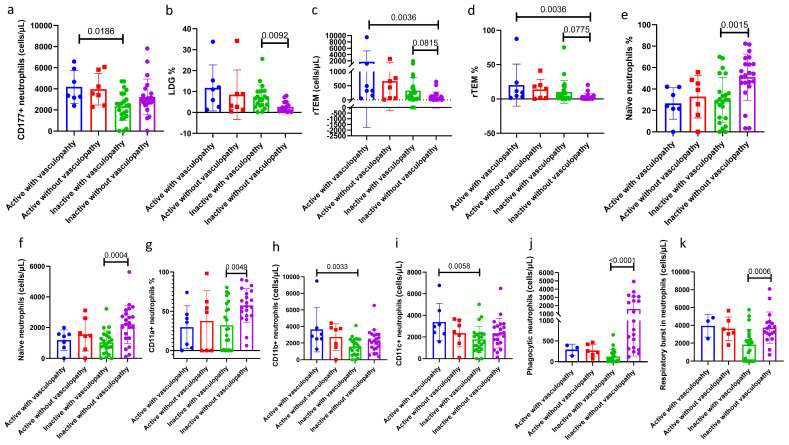
Phenotypic and functional neutrophil alterations in active vasculopathy. Patients with active vasculopathy have higher absolute activated neutrophil counts (3354 cells/μL [3163–5902] vs. 2450 cells/μL [1419–3543]; *p* = 0.0186), increased proportions of low-density granulocytes (LDGs) (6.87% [3.78–10%] vs. 2.42% [0.600–8.493%]; *p* = 0.0109), and a trend towards expanded reverse transendothelial migrating neutrophils (rTEM), in absolute numbers (131.3 cells/μL [30.26–766] vs. 45.25 cells/μL [14.04–145.1]; *p* = 0.0815) and percentage (4% [0.89–12.8%] vs. 1.47% [0.332–2.843%]; *p* = 0.0775). (**a**–**d**). Conversely, naïve neutrophils are reduced in vasculopathy number (908.9 cells/μL [371.2–1490] vs. 2260 cells/μL [1304–2955]; *p* = 0.0015). (**e**,**f**). Vasculopathy is associated with decreased CD11a expression in neutrophils (27.35% [0.094–60.45%] vs. 56.2% [44.3–74.05%]; *p* = 0.0049), while disease activity increases absolute counts of CD11b^+^ and CD11c^+^ neutrophils. CD11b+ (2828 cells/μL [2495–3829] vs. 1513 cells/μL [645–2435]; *p* = 0.0033) and CD11c+ (2965 cells/μL [2299–4441] vs. 1492 cells/μL [867–2283]; *p* = 0.0058). (**g**–**i**). Additionally, neutrophils from vasculopathic patients show reduced phagocytic capacity (51.57 cells/μL [0–210.3] vs. 882.8 cells/μL [316.3–2963]; *p* < 0.0001) and respiratory burst activity (1975 cells/μL [0–2755] vs. 3467 cells/μL [2837–4353]; *p* = 0.0006). (**j**,**k**). Kruskal–Wallis and Dunn’s post hoc tests, with Mann–Whitney U and Wilcoxon signed-rank tests were performed.

Active vasculopathic patients had more activated neutrophils (3354 cells/μL [3163–5902] vs. 2450 cells/μL [1419–3543]; *p* = 0.0186) and a higher proportion of LDGs (6.87% [3.78–10%] vs. 2.42% [0.600–8.493%]; *p* = 0.0109) than those without vasculopathy (Figure 3a,b). Reverse transendothelial migrating neutrophils (rTEM) were expanded in active vasculopathy both by count (323.4 cells/μL [153.4–1088] vs. 45.25 cells/μL [14.04–145.1]; *p* = 0.0036) and percentage (9.39% [3.23–23.8%] vs. 1.47% [0.332–2.843%]; *p* = 0.0036) compared to inactive patients without vasculopathy. Similar trends, though not significant, were observed in inactive vasculopathic patients (131.3 cells/μL [30.26–766] vs. 45.25 cells/μL [14.04–145.1]; *p* = 0.0815), (4% [0.89–12.8%] vs. 1.47% [0.332–2.843%]; *p* = 0.0775) (Figure 3c,d). Conversely, vasculopathic patients had reduced naïve neutrophils in number (908.9 cells/μL [371.2–1490] vs. 2260 cells/μL [1304–2955]; *p* = 0.0015) and percentage (29.8% [12.7–37.4%] vs. 53.5% [44.95–64.7%]; *p* = 0.0004) (Figure 3e,f). Integrin expression showed lower CD11a^+^ neutrophils in vasculopathy (27.35% [0.094–60.45%] vs. 56.2% [44.3–74.05%]; *p* = 0.0049), while active vasculopathic patients had increased CD11b^+^ (2828 cells/μL [2495–3829] vs. 1513 cells/μL [645–2435]; *p* = 0.0033) and CD11c^+^ (2965 cells/μL [2299–4441] vs. 1492 cells/μL [867–2283]; *p* = 0.0058) compared to inactive patients with vasculopathy (Figure 3g–i).

To address the functional implications of the expression profile we assessed diverse parameters. The functional analysis revealed that neutrophils from patients with vasculopathic phenotype had reduced phagocytic capacity (51.57 cells/μL [0–210.3] vs. 882.8 cells/μL [316.3–2963]; *p* < 0.0001) and decreased respiratory burst activity (1975 cells/μL [0–2755] vs. 3467 cells/μL [2837–4353]; *p* = 0.0006) (Figure 3j,k). To further elucidate the predictive role of these innate immunity features for vasculopathy development in IIM, univariate logistic regression identified disease activity associated with higher counts and MFI of CD11b^+^ and CD177^+^ neutrophils, total and subtypes of granulocytic MDSCs, and phagocytic monocytes. Vasculopathy-associated subpopulations included lower naïve neutrophils, CD11a^+^ neutrophils, immature granulocytic MDSCs, and reduced phagocytic neutrophils (Table 2). Multinomial logistic regression confirmed only naïve neutrophils (absolute counts; Chi^2^ = 20.76, *p* = 0.0001) and CD177^+^ neutrophils (absolute counts; Chi^2^ = 13.48, *p* = 0.0037) as independent vasculopathy predictors (Table 2).

**Figure 4:**
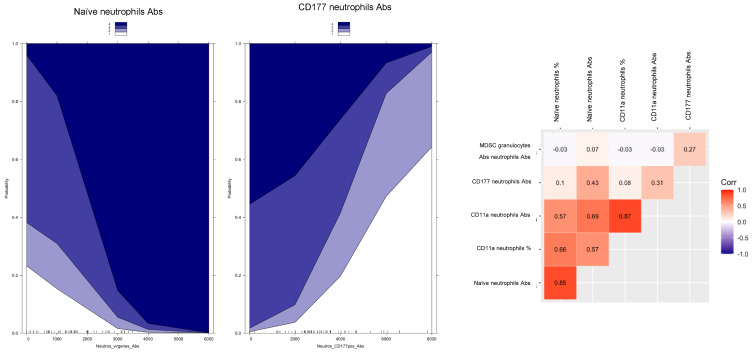
Neutrophil subset correlations by vasculopathy and disease activity status. Correlations among neutrophil subpopulations identified as predictors of vasculopathy in the multivariable analysis, according to operational subgroup definitions: (1) active with vasculopathy (white), (2) active without vasculopathy (periwinkle blue), (3) inactive with vasculopathy (cobalt blue), and (4) inactive without vasculopathy (indigo blue). Correlations among neutrophil subpopulations identified as predictors of vasculopathy in the multivariable analysis.

Propensity curves showed that decreased naïve and activated neutrophils correlated with increased vasculopathy risk (Figure 4). Correlation matrices revealed positive associations between CD177^+^ activated neutrophils and naïve neutrophils (r = 0.43; *p* < 0.05), and between CD11a^+^ neutrophils and naïve neutrophils (r = 0.69; *p* < 0.05) (Figure 4).

## 4. Discussion

Patients with vasculopathy show a distinct myeloid profile, including expanded LDGs and MDSCs (Arginase-1^+^/PDL1^+^), decreased naïve neutrophils, and impaired phagocytosis and respiratory burst, highlighting myeloid cells’ key role in vasculopathic lesion development. Notably, no significant differences were identified in the frequency of regulatory neutrophils between the compared groups.

While the inflammatory functions of neutrophils have been previously described, the characterization of their granulocytic subpopulations has been variable and inconsistent in the literature [11,29]. Recently, certain pre-activated proinflammatory phenotypes, such as LDGs, have been documented and associated with specific vasculopathic manifestations including calcinosis, cutaneous ulcers, the presence of anti-MDA5 autoantibodies [15], and enhanced capacity to form neutrophil extracellular traps (NETs) in anti-MDA5 and ILD IIM patients. In these cases, elevated circulating free DNA and the antimicrobial peptide LL37—structural components of NETs—were documented, especially those with cutaneous ulcers, hyperferritinemia, and increased KL-6 levels. These findings support the hypothesis that aberrant NET formation acts as a mechanism to induce and amplify tissue damage, particularly in target organs such as skin and lung [30]. These data are consistent with our findings of increased peripheral expansion of these subpopulations in patients with vasculopathy.

Reverse transmigrating neutrophils (rTEM) increase their peripheral blood concentration by 20–40% during enhanced microvascular permeability, mainly mediated by disruption of the chemotactic gradient caused by CXCL1 [19] leading to tissue damage. Although statistical significance was not reached, the biological gradient observed in rTEM suggests their role in distal migration to secondary sterile inflammation sites, especially relevant when evaluating vasculopathic involvement in IIM patients, as previously demonstrated by pulmonary, muscular, and cutaneous NET infiltration, particularly perivascular and uniquely in anti-MDA5^+^ patients [31].

In agreement with previous publications, the expansion of immature phenotypes such as granulocytic MDSCs was confirmed; these cells have correlated positively with disease activity, proinflammatory cytokines such as IL-6, IL-8, and GM-CSF, muscle enzymes including LDH, CPK, AST, and particularly the PDL1-expressing granulocytic MDSC subtype with the clinical variable of lung damage [22]. Moreover, consistent with earlier reports, the release of immature elements corresponds to the emergency granulopoiesis phenomenon, where these cells, although phenotypically “suppressive,” lack effective immunomodulatory mechanisms, either due to cellular exhaustion or functional immaturity [11,17,32]. This underscores the need to focus research on MDSCs and their role in systemic autoimmune diseases, particularly IIM.

The reduced phagocytic and respiratory burst capacity observed in clinically inactive patients with vasculopathy compared to those without this phenotype suggests persistent functional dysfunction in the neutrophil compartment even in the absence of overt clinical activity. The documented alterations in phagocytosis and respiratory burst may represent an “apoptosis escape” mechanism, especially relevant in tissue-experienced subpopulations such as rTEM. These reverse migrating cells classically express less caspase-3 but, contrary to what was shown in this study, produce higher concentrations of reactive oxygen species [17]. Our results demonstrated reduced phagocytosis and respiratory burst, similarly observed in MDSCs, where immature granulocytic precursors in peripheral blood are a hallmark of IIM patients, showing dysfunction secondary to chronic proinflammatory states and ineffective clearance of cellular debris and apoptotic bodies, as demonstrated in macrophages from IIM patients [33]. Our research group demonstrated an increased in phagocytosis in monocytes and neutrophils in systemic lupus erythematosus (SLE) patients, but a decreased respiratory burst intensity compared to healthy controls [34]. These observations were replicated by Gyimesi et al., reporting impairment of respiratory burst in other myeloid cells like monocytes after phagocytosis mediated by FcγRII and CR3 [35]. Therefore, we observed, for the first time, the same effect in inactive vasculopathic DM patients, which could be explained by a defective debris phagocytosis involving the proliferation and differentiation of myoblasts and impairment of redox capacity, especially in the glutathione peroxidase system [36]. More studies regarding these important questions are needed in order to unveil, the remaining major neutrophil function questions such as cytokine production, and organization of NETosis and apoptosis death programs, especially in IIM patients. Additionally, the expansion of dysfunctional subpopulations such as LDGs and MDSCs could interfere with the functionality of conventional neutrophils via immunoregulatory mediators like Arginase-1 and TGF-β [37]. Finally, remodeling of the immune microenvironment—particularly at the endothelial level—may alter key activation signals (such as those mediated by CD11a/CD11b), contributing to an incomplete or ineffective neutrophilic response [38]. Collectively, these findings support the hypothesis that vasculopathy represents a clinically silent but immunologically dysfunctional phenotype with potentially relevant prognostic and therapeutic implications.

Another neutrophil subpopulation with a proinflammatory phenotype is characterized by CD177^+^ expression, a surface glycoprotein differentially expressed on activated neutrophils. This molecule has been associated with increased affinity for PECAM-1, facilitating transendothelial migration and tissue infiltration, as well as with enhanced IL-17 production by neutrophils, contributing to the local inflammatory milieu [39]. Unexpectedly, multivariate analysis identified an inverse association between vasculopathy presence and absolute numbers of activated CD177^+^ neutrophils. This observation, seemingly contradictory to its known proinflammatory role, can be interpreted from several perspectives. First, the peak phase of neutrophilic activation and infiltration associated with CD177^+^ may occur transiently and early in the inflammatory process, followed by tissue redistribution, particularly in patients with advanced or chronic vasculopathic forms. Also, considering CD177^+^ has been linked to NET formation and endothelial damage amplification via the NLRP3 inflammasome, a lower circulating amount might reflect its mobilization to target tissues—such as skin or lung—where it exerts its pathological effect. This phenomenon has been previously described in murine models of ARDS, where tissue overexpression of CD177 is associated with severe pulmonary inflammation, while its inhibition reduces infiltration and proinflammatory mediator release [40,41]. Thus, in established vasculopathy, the reduction in this subpopulation does not necessarily imply a lesser pathogenic role but rather an advanced phase of inflammatory response or extravascular localization.

In parallel, multivariate analysis revealed a significant association between vasculopathy presence and a lower proportion of naïve neutrophils, suggesting an imbalance in myeloid compartment homeostasis. Naïve neutrophils, defined by their functional immaturity, are characterized by low activation capacity and limited proinflammatory mediator release; hence, their reduction could reflect a shift toward more activated, proinflammatory, and effector-capable neutrophil phenotypes, such as LDGs, MDSCs, and rTEM.

This decrease may also be interpreted as exhaustion of the basal neutrophil reservoir, resulting from excessive mobilization of precursors to activated peripheral compartments or accelerated maturation induced by systemic inflammatory signals—particularly those linked to vasculopathy such as hyperferritinemia and increased IL-6 and enhanced NETosis [15].

In this regard, the reduction in naïve neutrophils may represent an immunological imprint of sustained chronic inflammation, neutrophilic activation, loss of regulation, and diminished capacity to restore endothelial tolerance [19]. This finding reinforces the concept that, beyond total neutrophil counts, the balance between pro- and anti-inflammatory subpopulations may be decisive in the development of vascular damage in IIM patients.

Being an observational and cross-sectional study, causal relationships between the identified immunophenotypic alterations and vasculopathic manifestations cannot be established. The described associations should be interpreted cautiously and require longitudinal validation. Although the evaluated cohort allows identification of relevant patterns, the single-center nature introduces bias toward one ethnicity. Additionally, sample size may limit the generalizability of findings and the statistical power to detect subtle associations or differences in specific subgroups, such as those defined by antibody type.

Nevertheless, this is one of the few studies evaluating specific neutrophil and MDSC subpopulations in IIMs with direct clinical correlation to vasculopathy. This study provides valuable information on idiopathic inflammatory myopathy patients under active follow-up in a cohort allowing standardized clinimetric evaluation based on international consensus (IMACS), alongside an exhaustive analysis of granulocytic and monocytic myeloid subpopulations incorporating functional markers (PDL1, Arginase-1), activation markers (CD11a/b/c, CD177), and cellular functionality (phagocytosis and respiratory burst), allowing a comprehensive assessment of innate immune status. Activity-matched pairing isolated the effect of vasculopathy on the myeloid profile, reducing bias attributable to general inflammatory status and strengthening the internal validity of the associations found, thus providing translational evidence of possible immunological biomarkers implicated in vascular damage in IIMs.

## 5. Conclusions

In summary, patients with IIMs presenting a vasculopathic phenotype exhibit a distinctive immunological profile characterized by an expansion of proinflammatory myeloid subpopulations, including LDGs, Arginase-1^+^ and PDL1^+^ MDSCs, and rTEM. In contrast, there was a significant reduction in naïve neutrophils and a decrease in key effector functions such as phagocytosis and respiratory burst.

Multivariate analysis identified naïve neutrophils and activated CD177^+^ neutrophils as independent predictors of vasculopathy, suggesting that an imbalance between immunoregulatory and proinflammatory subpopulations may contribute to persistent endothelial damage in patients with IIMs.

These findings support a model of progressive myeloid activation, wherein loss of regulatory subpopulations alongside sustained and dysregulated neutrophil activation may promote the emergence of vasculopathic complications. The immunophenotypic profile described here may provide new tools for the immunological stratification of patients with IIM and could serve as a basis for developing prognostic biomarkers or therapeutic targets directed at neutrophil activation pathways.

## Figures and Tables

**Table 1:** Main demographic, clinical and serological features in patients with IIM according to vasculopathy and disease activity. Kruskal–Wallis test (Dunn’s multiple comparisons) and Mann–Whitney U test for quantitative variables; Fisher’s exact test for categorical variables.

Variable	Active with Vasculopathy Median (IQR) or Percentage	Active Without Vasculopathy Median (IQR) or Percentage	Inactive with Vasculopathy Median (IQR) or Percentage	Inactive Without Vasculopathy Median (IQR) or Percentage	*p*-Value
Age (years)	51 (44–58)	45 (39–53)	52.5 (45.5–61.75)	49 (31–59)	0.5604
Female (%)	71.4%	85.7%	86.9%	72.72%	—
Manual muscle test 8 (MMT8)	139 (133–149)	84 (42–148)	150 (150–150)	150 (150–150)	0.0011
CDASI activity	3 (0.5–18)	5.5 (0.25–12.25)	1 (0–3.5)	1 (0–3)	0.1289
CDASI damage	3 (0.5–9.5)	4 (0.75–5.75)	3 (0.25–5.5)	1.5 (0–2.7)	0.3768
Neutrophils (×10^3^/µL)	4570 (3390–7250)	3830 (3660–6370)	2910 (2555–4365)	4290 (3303–5283)	0.0129
Creatine kinase (U/L)	35 (31–179)	346 (47–1167)	56.5 (41–79.5)	114.5 (68.5–186.3)	0.0006
Aldolase (U/L)	3.6 (1.7–7.2)	9.4 (3.6–27.4)	3.4 (1.82–4.075)	3.75 (2.55–5.4)	0.0576
GOT (U/L)	15.9 (10.8–39.1)	50.8 (11.3–134.6)	17.15 (10.95–38.5)	20.85 (15.13–27.1)	0.6536
GPT (U/L)	34.9 (18.4–102.6)	20.25 (16.5–28.3)	20.25 (16.5–28.4)	20.95 (16.3–27.7)	0.3222
DHL (U/L)	229 (141–276)	332 (184–577)	159 (138–178)	157 (144–178)	0.0093
C-reactive protein (mg/dL)	0.4 (0.14–4.24)	0.16 (0.09–0.36)	0.15 (0.06–0.41)	0.23 (0.05–0.83)	0.5330
Ferritin (ng/mL)	173.8 (33.7–253.6)	501 (120–1311)	49 (18–95.7)	54.2 (23.65–95.75)	0.0044
Gamma globulins (g/dL)	2.8 (2.15–3.38)	2.78 (2.18–3.61)	3.04 (2.6–3.2)	2.7 (2.45–3.08)	0.6262
MSA/MAA antibodies	MDA5 (4/7), Ro52 (2/7), TIF1g, Jo1, PM75, Ku	Mi2 (2/7), TIF1g (2/7), Ro52 (3/7), PL12, Jo1, PM100	MDA5 (10/23), PL7 (2/23), PL12 (2/23), PM75 (2/23), NXP2 (2/23), Jo1, SRP, PM100, SAE	Mi2 (7/22), Neg (7/22), NXP2 (4/22), MDA5 (2/22), PL2, PM75, Ku, Ro52	—
Prednisone 1 mg/kg/d (%)	71.4%	100%	43.47%	14.2%	0.118
Prednisone cumulative dose (mg)	10 (0–50)	50 (10–60)	0 (0–5)	0 (0–0)	<0.0001
IVIG (%)	42.8%	57.1%	4.3%	0%	>0.9999
Plasmapheresis (%)	14.28%	0%	0%	0%	0.313
Cyclophosphamide (%)	14.28%	0%	4.3%	0%	0.4915
Rituximab (%)	71.42%	42.8%	26%	9.5%	0.1432
Mycophenolate mofetil (%)	71.42%	57.1%	34.78%	28.4%	0.5959
Tacrolimus (%)	66.6%	14.28%	8.6%	4.75%	0.6707
Methotrexate (%)	14.28%	14.28%	43.7%	61.9%	0.6010
Azathioprine (%)	0%	0%	17.39%	19%	>0.9999
Hydroxychloroquine (%)	57.1%	71.42%	52.17%	57.14%	>0.9999

IQR: Interquartile range; CDASI: Cutaneous Dermatomyositis Disease Area and Severity Index; GOT: glutamic-oxaloacetic transaminase; GPT: glutamic-pyruvic transaminase; DHL: lactate dehydrogenase; MSA: myositis-specific antibodies; MAA: myositis-associated antibodies.

**Table 2:** Innate immunity variables associated with vasculopathy in IIM patients.

**Outcome: Disease Activity**	**Chi^2^**	***p*-value**
Neutrophils CD11b+ (cells/µL)	4.58	0.032
Neutrophils CD11b+ (MFI)	6.70	0.0096
Neutrophils CD177+ (cells/µL)	5.86	0.015
Neutrophils CD177+ (MFI)	4.13	0.042
Granulocytic MDSCs (%)	5.38	0.020
Granulocytic MDSCs (cells/µL)	6.25	0.012
Mature granulocytic MDSCs (%)	6.51	0.010
Mature granulocytic MDSCs (cells/µL)	8.28	0.004
Immature granulocytic MDSCs (cells/µL)	7.28	0.007
Phagocytic monocytes (cells/µL)	3.98	0.046
**Outcome: Vasculopathy**	**Chi^2^**	***p*** **-value**
Naïve neutrophils (%)	8.05	0.0046
Naïve neutrophils (cells/µL)	9.88	0.0017
Neutrophils CD11a+ (%)	6.62	0.010
Neutrophils CD11a+ (cells/µL)	7.56	0.006
Immature granulocytic MDSCs (cells/µL)	6.36	0.012
Phagocytic neutrophils (cells/µL)	5.22	0.022
**Outcome: Vasculopathy (Multivariate)**	**Chi^2^**	***p*** **-value**
Naïve neutrophils (cells/µL)	20.76	0.00012
Neutrophils CD177+ (cells/µL)	13.48	0.0037
**MDSCs**: Myeloid-derived suppressor cells		

## Data Availability

The datasets used and/or analyzed during the current study are available from the corresponding author on reasonable request.
